# Comparison of three different methods of internal sinus lifting for elevation heights of 7 mm: an ex vivo study

**DOI:** 10.1186/s40729-017-0103-5

**Published:** 2017-09-04

**Authors:** Aghiad Yassin Alsabbagh, Mohammed Monzer Alsabbagh, Batol Darjazini Nahas, Salam Rajih

**Affiliations:** 1 Department of Periodontology, Damascus University Dental School, Damascus, Syrian Arab Republic; 2 Department of Orthodontics, Damascus University Dental School, Damascus, Syrian Arab Republic; 3 Temple university, Philadelphia, USA

**Keywords:** Maxillary sinus, Schneiderian membrane, Sinus floor elevation, Balloon elevation, Elevation height, CAS kit

## Abstract

**Background:**

Various techniques are available for elevating the sinus membrane. The aim of this study is to evaluate three methods of indirect sinus floor elevation regarding elevation heights of 7 mm on the outcomes of membrane perforation, length of perforation, and time required to perform the procedure**.**

**Methods:**

Three different methods for indirect sinus lifting, bone added osteotome sinus floor elevation (BAOSFE), sinus floor elevation with an inflatable balloon, and crestal approach system (CAS kit) from OSSTEM, were assessed for their ability to lift the sinus without causing laceration of the Schneiderian membrane. The study was performed on 18 freshly slaughtered sheep heads (36 sinus lifts were done, 12 for each method). CBCT images of the heads were taken to assess the best location for the sinus lift. Then, the heads were bisected and the membrane was exposed from the medial aspect. After that, each method was performed. The intended elevation height was 7 mm. If the 7 mm were not reached, the maximum height of elevation was measured.

**Results:**

The method used was significantly associated with the occurrence of perforation (*p* value = 0.014) where BAOSFE was associated with the largest number of perforations (58.4%, *n* = 7) compared to 8.3% and 8.3% for the balloon and CAS kit methods, respectively. The odds ratio for perforation occurrence from BAOSFE compared to the CAS kit was significant (OR = 0.091, *p* = .022). No significant odds ratio was found for the balloon method compared to CAS kit. Additionally, the method used was significantly associated with time of operation and with the length of perforation (*p* value < 0.001) where CAS kit required the longest time and BAOSFE caused the biggest perforations.

**Conclusions:**

The study shows that both the balloon and the CAS kit were superior to the BAOSFE in terms of safety in elevating the sinus membrane. Further, in vivo studies have to prove these findings.

## Background

More than half of the implants placed in the posterior maxilla require sinus floor elevation (SFE) [1]. The need for this procedure is explained by continuous ridge resorption in an apical direction after tooth extraction combined with progressive sinus pneumatization in addition to poor bone quality that is frequently seen in the maxilla [2].

Sinus membrane perforation is considered the most common complication during sinus floor elevation procedures, and its percentage varies according to the method used. Perforations happen either while fracturing the floor of the sinus or during the elevation of the mucosa [3, 4].

Crestal approach to the sinus kit (CAS kit) was introduced by OSSTEM implants (Osstem Implant Co., Busan, Korea) as a safe and effective method for sinus elevation with the advantage of using a reamer (the CAS drill) to perform the osteotomy in a conical shape and break the bony floor; however, only one questionnaire that assessed the satisfaction of dentists using the CAS KIT is available in the literature on this method [5]. Using an inflatable balloon for indirect sinus floor elevation has been shown to be successful in elevating the mucosa for elevation heights of up to 10 mm [6, 7]. However, few studies in the literature compared this technique to others.

Lopez-Nino et al. studied the lamb as an ex vivo model for training in sinus floor elevation and concluded that the model is useful because of the similarities in the thickness of the lateral wall of the maxillary sinus and the thickness of the Schneiderian membrane between the models and the human standards [8].

Cone beam computed tomography (CBCT) can precisely visualize the sinus complexity in 3D, with low irradiation to the patient. In implant dentistry, recent guidelines recommend the use of CBCT for three-dimensional treatment planning, especially prior to SFE for evaluating both residual alveolar and sinus conditions [9, 10].

Therefore, the two working hypotheses of our study were “the CAS-Kit is safer than BAOSFE in breaking the sinus floor and the balloon is safer than BAOSFE in elevating the Schneiderian membrane” for elevation heights of 7 mm.

## Methods

### The sample

**Fig. 1:**
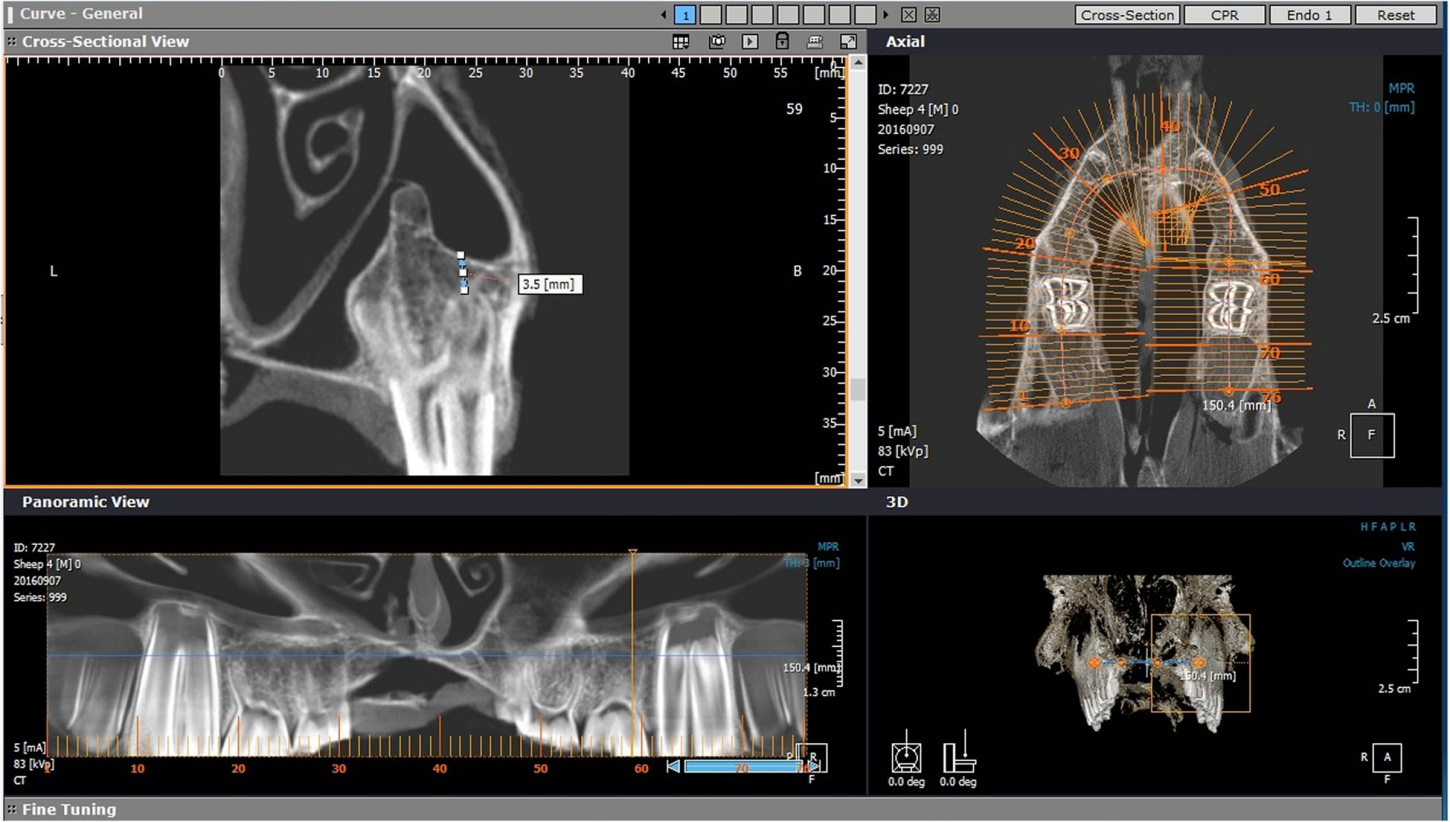
Determination of the remaining bone height (RBH) on the CBCT image

To achieve our purposes, an experimental ex vivo study was carried. This research project was approved by the University of Damascus Local Research Ethics Committee (UDDS-3045PG.) and was funded by the Damascus University Postgraduate Research Budget (97687027834DEN). The sinus floor elevations were done on 18 bisected heads of lambs aged between 6 and 12 months that were slaughtered in a maximum of 4 h before the procedures began. CBCT images of the heads were taken using the Picasso® Pro CBCT system (Vatech™, Seoul, South Korea) set at a voxel size of 0.2 mm, tube current of 5 mA, tube voltage of 83 KV with gray scale of 16 bit per pixel. A standardized position of the lamb’s heads was maintained by the correct head orientation in accordance with the 3D intersecting planes of the red beam. Then, the images were analyzed for the best location to perform the sinus elevation where remaining bone height (RBH) is less than 5 mm on 3DOnDemand® programme (CyberMed, Finland) (Fig. 1). The RBH was measured from the apical tip of the buccal root on the third premolar which will be extracted to the floor of the sinus. The sample was randomized by generating random numbers using Research Randomizer software (http://www.randomizer.org/) [11] making sure that the same method was not done on the same lamb twice.

### Visual assessment

**Fig. 2:**
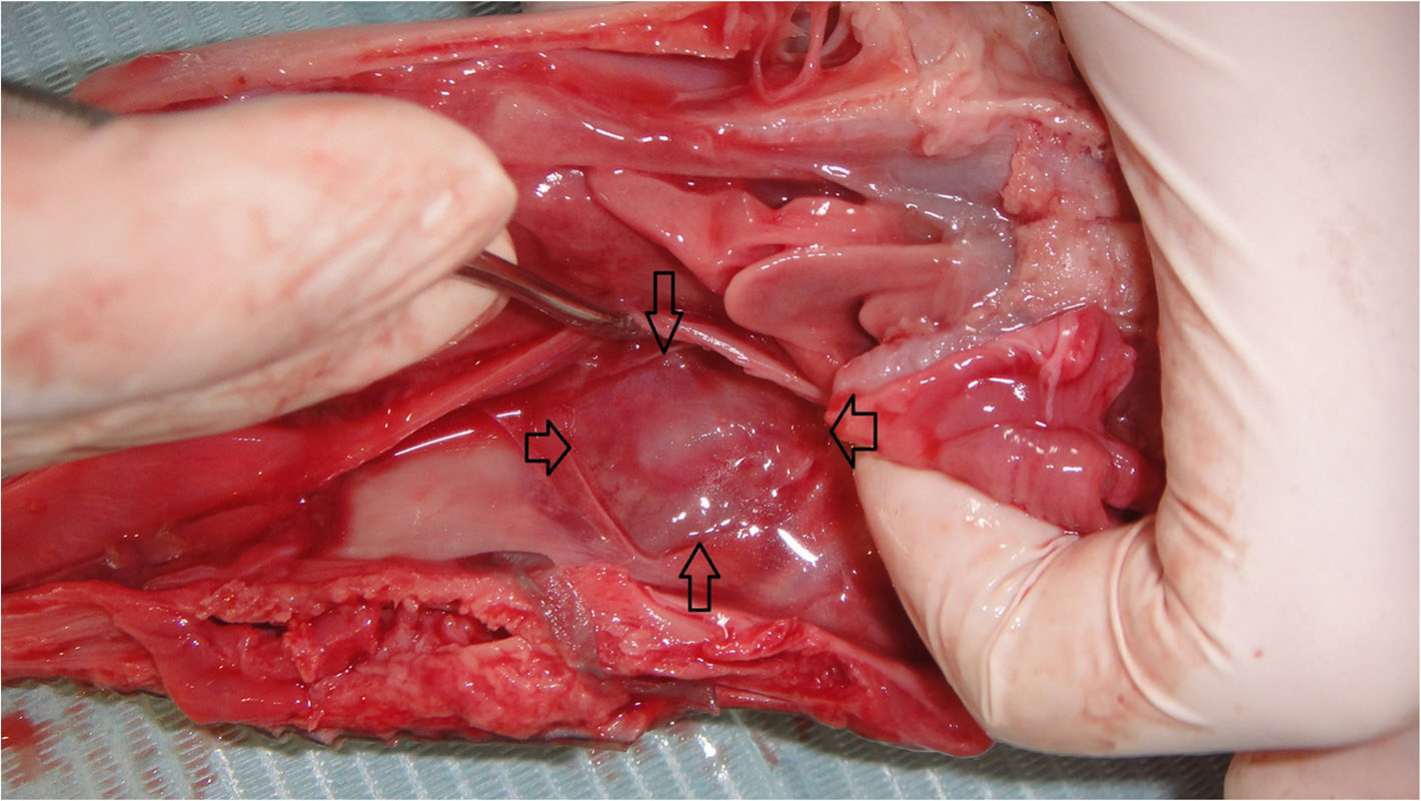
The exposed mesial aspect of the sinus

After the extraction of the third premolar, the mesial side of the sinus was exposed (Fig. 2) in order to check the sinus for any perforations. The elevation height was measured using a depth gauge, and the intended elevation height was 7 mm. If this height was not achieved, the maximum elevation was recorded. When a perforation of the membrane was present, its length was measured using a periodontal probe.

### Sinus floor elevation methods

#### BAOSFE

Bone blocks were harvested from the lamb’s head and made into soft bone particles using ACE bone mill®(ACE surgical Supply Co., Inc., Brockton, Ma, USA). For this technique, the osteotomy started with a pilot drill for 2 mm followed by burs with increasing diameter up to 3.2 mm. Then, osteotomes (FRIALIT-2 bone expander, Friadent, DENTSPLY Implants) were used to expand the osteotomy and to break the sinus floor after the addition of bone. The 4.5 mm osteotome was used to break the sinus floor and push continuous insertions of bone particles. Every use of the osteotome to pack the bone is expected to lift the sinus membrane for 1 mm [12].

#### Balloon sinus lift

**Fig. 3:**
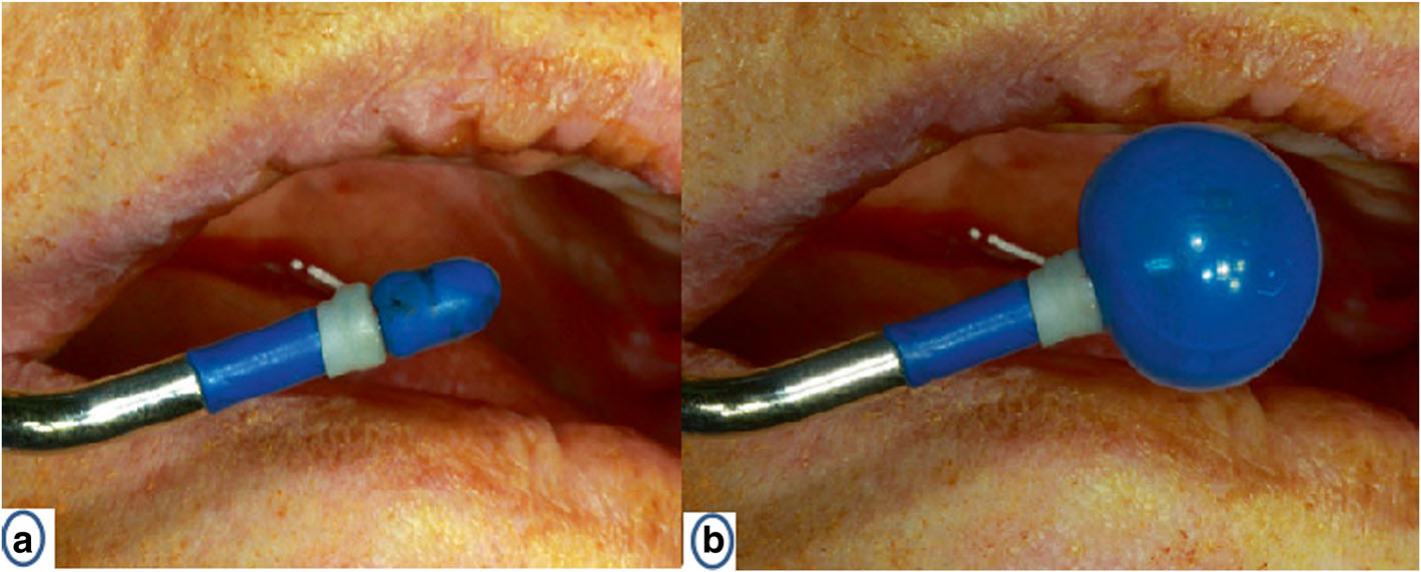
**a** The balloon in a resting position. **b** The inflated balloon [12]

**Fig. 4:**
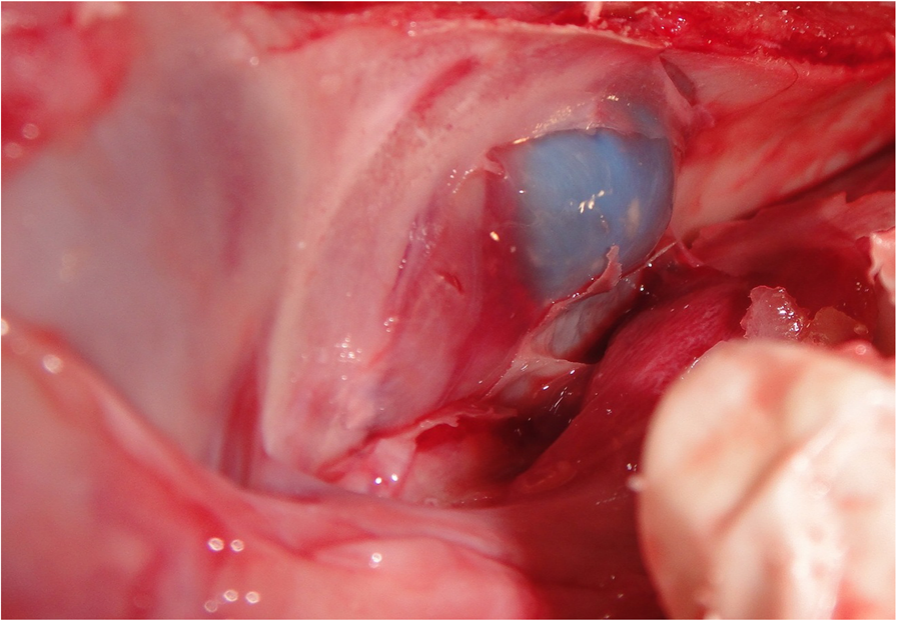
The inflated balloon while elevating the sinus membrane (The balloon is seen from the medial.)

This approach starts like BAOSFE. The osteotomy is enlarged to 5.0 mm before the balloon (Zimmer Sinus Lift Balloon, Zimmer Dental Inc., California, USA) is inserted (Fig. 3). The sinus floor was broken with the 5 mm osteotome after the addition of bone. The sleeve of the balloon was inserted 1 mm beyond the sinus floor. The saline was injected slowly from the syringe into the balloon, so the balloon would inflate progressively (Fig. 4). The balloon was deflated, and the desired elevation was checked if the elevation was not reached. The balloon was inserted again, and the process is repeated until the desired 7 mm are reached. One cubic centimeter of saline is expected to lift the membrane for 6 mm [13].

#### CAS kit

**Fig. 5:**
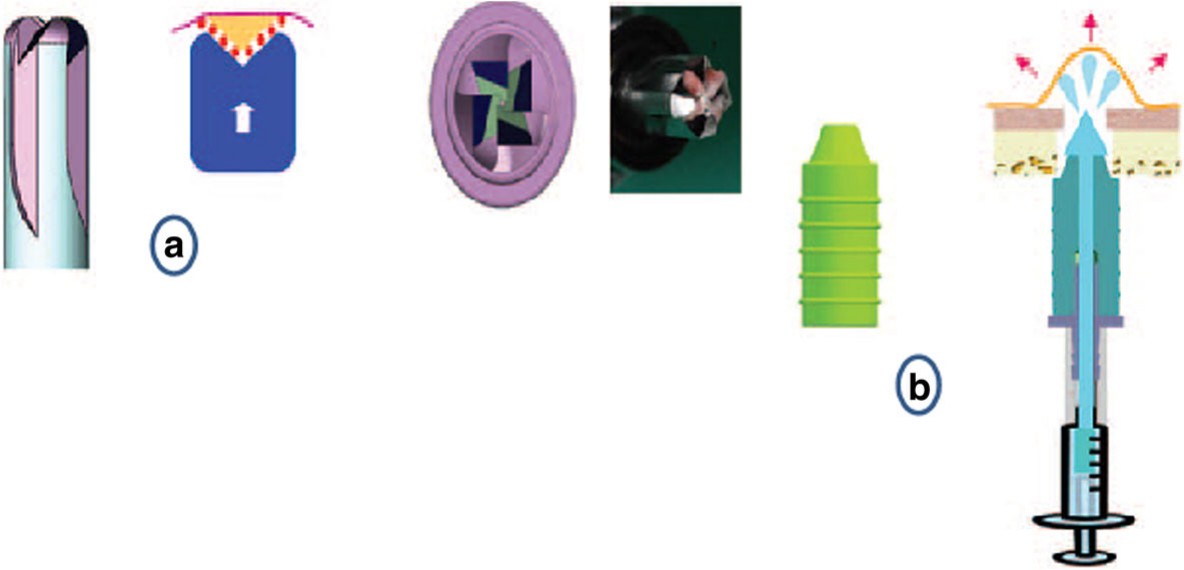
**a** The CAS drill has four blades and an inverse conical shape. **b** The hydraulic lifter

**Fig. 6:**
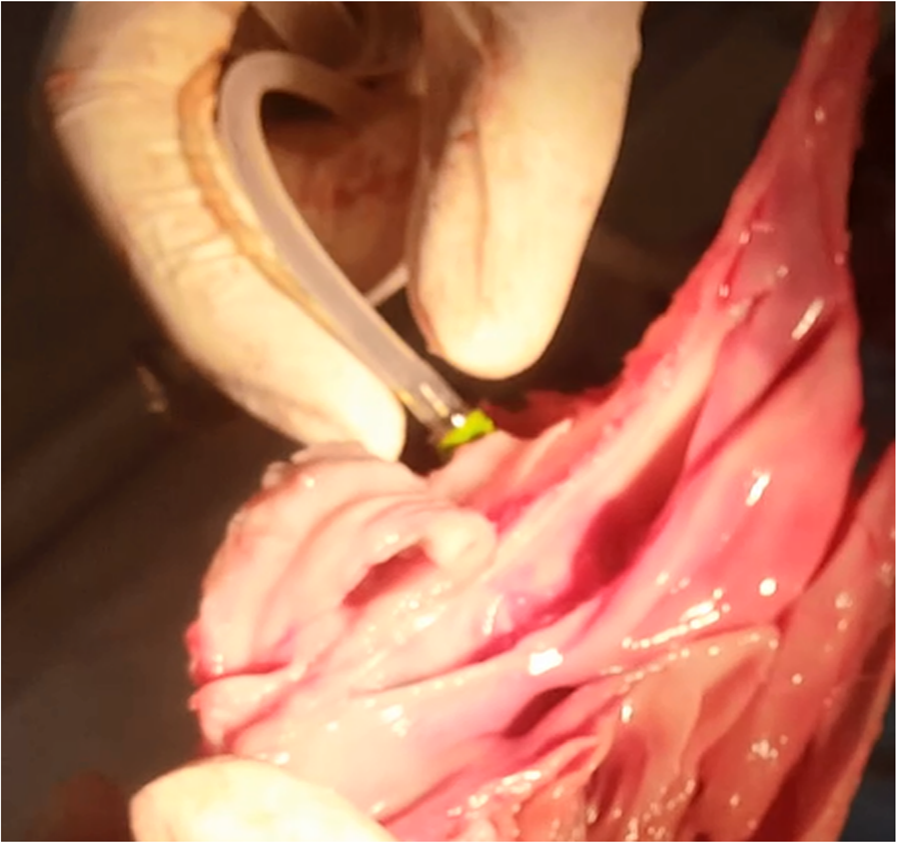
The hydraulic lifter stabilized in the osteotomy before injecting the saline

The CAS kit consists of a set of safe end drills, metal stoppers, a depth gauge, a hydraulic lifter, bone graft carrier, condenser, and a bone spreader (Fig. 5). The procedure started with a 2-mm twist drill. The drills were used to enlarge the osteotomy and are stopped 1 mm short of the sinus floor. The sinus floor was broken with the 3.6 mm bur without going through the floor; a depth gauge was used to check the membrane integrity and to slightly lift the membrane. Then, the hydraulic lifter was inserted and stabilized (Fig. 6) and the saline solution is injected. 0.30 mL can elevate the membrane up to 3 mm [5]. The saline is drown out then injected again until the desired elevation is reached.

### Statistics

Chi-square test was used to test the association between the three techniques and the occurrence of perforation whereas ANOVA (analysis of variance) was used to assess the association between method used and the two outcomes of the length of the perforation and the time of operation. Logistic regression of method used on the occurrence of perforation was employed to evaluate the odds of perforation for each method. *P* values equal to or smaller than .05 were considered to be significant. All calculations were made using SPSS version 16 for Windows (SPSS®, Chicago, IL, USA).

## Results

For the entire sample, the mean perforation length was (0.711 mm, SD = 1.4) and the mean time required to perform the procedure was (5.65 min, SD = 2.26), and out of the entire sample (*N* = 36), perforations happened in nine cases for a percentage of 25%.

Chi-square test showed a significant association between method used and the occurrence of perforation (chi-square statistic = 8.585, df = 2, *p* value = 0.014), as shown in Table 1. Also, ANOVA test showed a significant association between method used and the length of perforation (*F* = 11.031, df = 2, 33, *p* value < 0.001) where the BAOSFE caused the largest mean length of perforations (3.42 mm) followed by the CAS kit and the balloon (0.5, 0.5 mm). As for the time required to perform the procedures ANOVA test showed a significant association between method used and the time required to perform it (*F* = 1221.2, df = 2,33, *p* value < 0.001); CAS kit required the longest time (8.486 min) followed by the balloon then BAOSFE (5.393, 3.073 min) (Table 1).

**Table 1:** The association between the methods used the following variables: occurrence of perforation, length of perforation, and the time of operation

	BAOSFE	BALLOON	CAS kit	Total	Stats	*p* value
Occurrence of perforation	7 (58.4%) *N* = 12	1 (8.3%) *N* = 12	1 (8.3%) *N* = 12	9 (25%) *N* = 36	× 2 = 8.585^a^	0.014
Length of perforation (mean)	3.42 mm	0.5 mm	0.5 mm	0.711 mm	*F* = 11.031	0.0001
Time of operation (mean)	3.073 min	5.393 min	8.486 min	5.651 min	*F* = 1221	0.0001

*F* ANOVA test

^a^Chi-square test

Table 2 shows the results of logistic regression of method used on the occurrence of perforation, the odds ratio showed significant differences between the balloon technique and the BAOSFE (OR = 0.091, *p* value = 0.022), and between the CAS kit and the BAOSFE (OR = 0.091, *p* value = 0.022); however, no significant differences were found between the balloon and the CAS kit (OR = 1,0, *p* value = 1). It should be noted that the CAS kit was only able to lift the membrane for a maximum of 5 mm.

**Table 2:** The results of logistic regression of method used on the occurrence of perforation

Methods	BAOFSE		BALLOON		CAS kit	
Number of cases	12		12		12	
Number of perforations	7		1		1	
Percentage	58.4%		8.3%		8.3%	
Comparison of methods regarding perforations (odds ratio)						
	Balloon\BOAFSE		Balloon\CAS kit		CAS kit\BAOFSE	
Odds ratio	0.091		1		0.091	
*p* value	0.022		1		0.022	
Confidence interval	Lower	Upper	Lower	Upper	Lower	Upper
	1.437	160.972	0.55	18.085	1.437	160.972
Reference group	BAOSFE		BAOSFE		CAS kit	

## Discussion

Although the lateral sinus floor elevation is a proven clinically successful technique [14], the indirect SFE approach is favorable among clinicians because it does not require a second surgery site and hence cause less trauma and discomfort for the patient [14–16]. However, this method has its drawbacks, such as a higher risk of membrane perforation, a decreased space for using surgical instruments, and limitation in elevation heights when using the conventional techniques [3, 16, 17].

The osteotome technique originally described by Tatum 1994 has been shown microscopically to elevate the sinus floor for 5 mm without causing perforations [18]. Thus, this technique should not be used when the intended elevation height is more than 5 mm [19]. Therefore, a need for transalveolar approach that can elevate the membrane safely and for elevation heights greater than 5 mm has risen, Tatum described a modified approach to his osteotome technique in which bone particles are pushed in the sinus. The addition of bone will prevent direct contact between the instruments and the membrane [20]. Recently, many methods for SFE have been described as an alternative for the osteotome technique. Most of this techniques fall under two categories: using an inflatable device such as a balloon or using hydraulic pressure, both of which have been shown to reduce the rate of membrane perforation [6, 7, 13, 21, 22]. Soltan and Smiler described the use of the balloon and concluded that it is a highly successful and easy to perform procedure [6]. Recently, many systems have been developed which rely on hydraulic pressure to lift the sinus mucosa including the Jeder-System (Jeder GmbH, Vienna, Austria) which consists of a drill with a chamber which is filled with saline solution. After the initial drilling is done, the drill is connected to a pump that produces high hydraulic pressure; the pressure is used to break the sinus floor and to lift the membrane [23]. Also, OSSTEM implants introduced the CAS kit as a method for preparing the osteotomy and elevating the membrane through hydraulic pressure.

Using a reamer instead of the osteotomes for breaking the sinus floor has the advantage of creating a thin bone shell that prevents direct contact between the drill and the Schneiderian membrane [24]. Moreover, using a reamer has been shown to cause less discomfort and nausea when compared to the osteotome technique as a result of the constant tapping of the osteotomes [25]. As a result, the CAS kit has the advantage over the BAOSFE and the balloon in preparing the osteotomy and breaking the sinus floor safely and with less complications. Moreover, it was noted during our study that using a drill gives better feedback to the surgeon when breaking the sinus floor compared to the osteotome.

However, in our study, the CAS-kit was able to lift the membrane for a maximum of 5 mm. We believe that the saline pressure injected through the hydraulic lifter from a syringe is small and decreases gradually after leaving the lifter, whereas a study on the Jeder system showed a height gain of (9.2 ± 1.7 mm). This could be attributed to the high hydraulic pressure from the Jeder pump which is a machine that control the hydraulic pressure [23]. On the other hand, in our study, the balloon was able to lift the membrane for 7 mm in all cases; therefore, the balloon was better in elevating the mucosa.

Our study compared between three techniques for SFE for elevation heights of 7 mm. The 7 mm elevation height was chosen as a previous study by Stelzle et al. 2011 showed that BAOSFE caused perforations in the mucosa in all samples for perforation heights of 10 mm [7]. Therefore, we tried to set a threshold that might be achieved with internal sinus lifting techniques and be feasible in clinical practice. Perforations were checked using the three different methods: the mesial window, using a depth gauge, and the injection of saline solution through the osteotomy, which allowed for accurate recording of perforations.

The BAOSFE technique caused perforations in the membrane in 7 out of 12 cases with a percentage of 58.4. This result is consistent with many previous studies which state that this technique has a high rate of perforations when the RBH is less than 5 mm [2, 7, 26]. Also, all the perforations happened during the elevation process; however, this percentage is different than that reported by Steltzle (100%) in a similar study as the intended elevation height was less by 3 mm in our study [7].

For the balloon technique, only one perforation happened during the elevation process and the balloon was able to lift the membrane for 7 mm in all successful cases. This result supports various studies that showed a high success rate for this technique [6, 7, 13]; however, the osteotomy should be enlarged to 5 mm before inserting the balloon and this might limit the indications for this technique in thin ridges.

The CAS kit caused perforation of the Schneiderian membrane in one of the 12 cases (8.3%) which happened during the osteotomy. This is the first study to our knowledge to assess the CAS kit form OSSTEM implants since we found one published article that was a questionnaire sent to dentists who used the system to assess their satisfaction with the CAS kit, The study reported a membrane perforation rate of 4.1%. This percentage is smaller than that reported in our study (8.3%); however, we believe that our method of checking perforations is more accurate. Also, the difference in sample size may have contributed to the outcome [5].

## Conclusions

Within the limitation of this study and that of an ex vivo study, we can accept our hypotheses that the balloon is better than the BAOSFE in elevating the membrane mucosa and the CAS kit is better than the BAOSFE in preparing the osteotomy and breaking the sinus floor for elevation heights of 7 mm. Further, in vivo studies need to be taken to prove these findings.
