# Correction to: Comparison of three different methods of internal sinus lifting for elevation heights of 7 mm: an ex vivo study

**DOI:** 10.1186/s40729-020-00238-2

**Published:** 2020-06-20

**Authors:** Aghiad Yassin Alsabbagh, Mohammed Monzer Alsabbagh, Batol Darjazini Nahas, Salam Rajih

**Affiliations:** 1 Department of Periodontology, Damascus University Dental School, Damascus, Syrian Arab Republic; 2 Department of Orthodontics, Damascus University Dental School, Damascus, Syrian Arab Republic; 3 Temple University, Philadelphia, USA

**Correction to: International Journal of Implant Dentistry (2017) 3:40**

**https://doi.org/10.1186/s40729-017-0103-5**

Following publication of the original article [1], the authors would like to point out that it was Summers 1994 who originally described the osteotome technique. Regarding Figure 5 they would like to amend that it is based on the 07/2014 edition of the CAS-KIT manual.
